# Impact of COVID‐19 pandemic on trauma mortality patients: A retrospective observational study in an Iranian level 1 trauma center

**DOI:** 10.1002/hsr2.1883

**Published:** 2024-02-13

**Authors:** Seyyed HamidReza Ayatizadeh, Roham Borazjani, Reza Fereidooni, Kazem Jamali, Hossein Abdolrahimzadeh Fard, Reza Homaeifar, Leila Shayan, Zohreh Saadatjoo, Shahram Paydar

**Affiliations:** ^1^ Student Research Committee Shiraz University of Medical Sciences Shiraz Iran; ^2^ Trauma Research Center, Rajaee (Emtiaz) Trauma Hospital Shiraz University of Medical Sciences Shiraz Iran; ^3^ Health Policy Research Center, Institute of Health Shiraz University of Medical Sciences Shiraz Iran

**Keywords:** COVID‐19, ISS, RTS, trauma, trauma scores, TRISS

## Abstract

**Background and Aims:**

The COVID‐19 pandemic has reshaped the epidemiology of various clinical conditions, including trauma which is closely tied to social policies. This study examines and compares the characteristics of trauma mortality patients, and their initial prognostic trauma scores, in the pre‐pandemic and pandemic periods.

**Methods:**

We conducted a retrospective observational study involving patients who passed away at a level 1 trauma center from July 23, 2018, to February 19, 2020 (prepandemic), and from February 20, 2020, to September 22, 2021 (pandemic). A subgroup analysis that matched 12 of the same months of the year in the two periods was also done. Patients who arrived deceased or passed away immediately upon arrival were excluded from data analysis. We collected and analyzed demographic and clinical data, employing the Abbreviated Injury Score (AIS), Injury Severity Score (ISS), Revised Trauma Score (RTS), and Trauma and ISS (TRISS) to compare initial prognoses.

**Results:**

Our study encompassed 1128 patients, with 529 in the prepandemic group and 599 in the pandemic group. Demographic characteristics showed no significant differences in the number of patients in the two periods. Motor vehicle accidents remained the predominant injury mechanism in both periods. While the mean ISS increased insignificantly (22.80 vs. 22.91, *p* = 0.902), the mean RTS decreased (6.32 vs. 5.82), and TRISS increased (23.97% vs. 28.93%) during the pandemic (*p* < 0.05). Hospital length of stay decreased in the pandemic period (15.57 vs. 12.54 days, *p* < 0.05). Subgroup analysis revealed increased ISS, decreased RTS, and increased TRISS during the pandemic (*p* < 0.05).

**Conclusion:**

In conclusion, while overall demographics and injury mechanisms remained virtually unchanged, trauma patients during the pandemic displayed worse estimated clinical prognoses, particularly in physiological trauma scores. The heightened mortality rate was attributed to poorer clinical conditions of patients.

## 1 BACKGROUND

COVID‐19 emerged in late 2019 as a novel viral pneumonia and within a few months reached global pandemic status.^1^ Various parts of healthcare systems have been impacted by the pandemic.^2^ The total number of patient admissions in the field of general surgery has experienced a decline.^3^ The significance of the interaction between COVID‐19 and trauma lies in that during a pandemic causing a healthcare crisis, the overcrowding of hospitals and allocation of healthcare resources to the new disease can potentially undermine patient care.^4^, ^5^ Mitigation policies such as lockdowns and social distancing implemented during the COVID‐19 pandemic can influence and transform the epidemiology of certain diseases, with injuries being especially impacted.^6^, ^7^

Trauma has remained a significant healthcare concern worldwide, with a tremendous impact on the global burden of disease.^8^ Compared to high‐income countries, in low‐ and middle‐income countries trauma represents a more substantial public health challenge.^9^ The effect of COVID‐19 on trauma patterns and care has been studied in several different locations. A scoping review of literature by Waseem et al. showed that the overall number of trauma cases, sports injuries, and motor vehicle accidents (MVAs) decreased during the studied COVID‐19 periods,^10^ and the study by Yasin et al. also demonstrated a decreased number of MVAs worldwide.^11^ Changes in injury patterns amid the pandemic could affect the clinical outcomes of trauma patients, as both the severity of trauma and the allocation of healthcare resources and personnel may change. Comparing the injury severity and mortality of trauma patients in pre‐COVID‐19 and COVID‐19 eras, some studies have shown no significant change,^12^, ^13^, ^14^, ^15^ while others report an increasing trend,^16^, ^17^ although this area remains understudied. A study by Driessen et al. demonstrated that COVID‐19 negatively impacted trauma care, with patients being less often admitted to the intensive care unit (ICU).^18^ At low resource settings, increased adjusted trauma‐associated mortality has been noted and pre‐hospital care, transportation, intensive care utilization, and shortage of nursing staff were recommended as bottlenecks for interventions.^19^

Benchmarking the performance of trauma centers remains a pertinent tool in evaluating and improving the quality of care and patient outcomes.^20^ The early determination of injury severity and prognosis of trauma patients has a key role in allocating surgical and intensive care resources.^21^, ^22^, ^23^ Different scoring systems have been designed and validated to standardize the assessment of injury severity and mortality prediction in trauma patients and benchmark the performance of trauma centers.

Our study aims to report on and compare the number, injury severity, injury mechanism, demographic characteristics, and retrospective clinical prognosis of trauma‐related mortality patients (based on the patients' trauma scores) in periods before and during the COVID‐19 pandemic. We also aim to determine if any changes in mortality were congruent with the changes in the clinical prognoses of patients, as determined by their trauma scores; if the mortality patients during any phase exhibit more adverse trauma scores, it suggests that the mortality rate can be attributed more to the severity of the condition than to the quality of trauma care.

## 2 METHODS

This is a retrospective observational study comparing the characteristics of trauma mortality cases including the number, injury types, and prognostic trauma scores. This manuscript adheres to the Strengthening the Reporting of Observational Studies in Epidemiology (STROBE) guideline.^24^

### 2.1 Study settings and participants

The study population was trauma mortality patients who were admitted to Shahid Rajaei (Emtiaz) Hospital (a level 1 trauma center in Fars province, south‐central Iran) in the prepandemic and pandemic periods. The 19 months before the official announcement of the first COVID‐19 cases in Iran (from July 23, 2018 to February 19, 2020) indicate the pre‐pandemic period and the 19 months following the official announcement (from February 20, 2020 to September 22, 2021) indicate the pandemic period studied. The inclusion criteria encompassed individuals who passed away while under admission due to trauma during the specified timeframes. Exclusion criteria applied to cases where clinical records were incomplete or unavailable. Patients who arrived deceased were also excluded from analysis for comparing the trauma scores; however, their descriptive statistics are reported.

### 2.2 Subgroup analysis

For period subgroup analysis, 12 consecutive months from the matching times of year in the prepandemic period and pandemic period were selected (prepandemic from September 23, 2018 to September 22, 2019, and pandemic from September 22, 2020 to September 22, 2021). This was done to account for the different incidences and epidemiology of trauma at different times of the year and to also exclude the first 7 months of the pandemic when it had not reached its full effects and to allow for stabilization and adaptation of the healthcare system.

### 2.3 Trauma scores

Abbreviated injury scale (AIS), Injury Severity Score (ISS), Revised Trauma Score (RTS), and the combined system of Trauma and Injury Severity Scoring (TRISS) remain the most widespread and validated scoring systems in trauma, despite limitations.^25^ AIS is a standardized scale for rating injury severity in each anatomic area and system, ranging from 0 to 6 and divided into 6 anatomic categories.^26^, ^27^ ISS, developed by Baker et al. in 1974, focuses on the anatomic distribution and severity of injuries and has a range of 1−75. ISS is calculated as the sum of the squares of a patient's three highest AIS scores, with patients having any AIS of 6 in any organ system automatically assigned an ISS of 75.^28^, ^29^ Developed by Champion et al., RTS has a range of 0−7.841 and assesses the physiologic status of a trauma patient by using the Glasgow Coma Scale (GCS), systolic blood pressure (SBP), and respiratory rate.^30^ TRISS which was originally developed by Boyd et al combines ISS and RTS with the patient's age and injury mechanism type (blunt vs. penetrating) to estimate their probability of mortality.^31^, ^32^ The regression coefficients established in 1995 and derived from the Major Trauma Outcome Study (MTOS) database were used in the calculation of TRISS.^33^ AIS of ≥3 was defined as a major injury in our study. ISS of <16 is conventionally defined as minor trauma, and ISS ≥ 16 as major trauma. In this study, ISS is subcategorized into <16, 16−24, and >24 groups. Furthermore, RTS of <4 and TRISS of ≥50% also denote major trauma in our study. These cutoffs for RTS and TRISS were selected at the mid‐point of possible score ranges for practicality and clinical relevance.

### 2.4 Data collection

The total number of admitted patients in our center and their cause of admission were extracted from the hospital information system (HIS). All patients admitted due to acute trauma whose death was registered in Shahid Rajaei Hospital (Emtiaz) in the two periods of interest were included (a total of 1129), and one patient was later excluded due to an outlier age of five. Patients who were readmitted for any complications or continuation of treatment after the initial hospital course were not included in our study. Demographic and initial clinical data (including AIS) were recorded by emergency department (ED) physicians and nurses (house staff and trainees). The sources of data on injury mechanisms were emergency medical services (EMS) personnel, patients, or their accompanying persons. These data were then extracted from Shahid Rajaei center's HIS and placed into a Microsoft Excel spreadsheet. Collected data included the patient name and admission number, age (further categorized into <18, 18−65, and >65 groups), gender, mechanism of injury, mode of arrival, and transportation (primary EMS/HEMS arrival, primary personal arrival, transferred from other facilities by EMS/HEMS), admission time and date, death time, date and ward, AIS scores, ED arrival and exit time systolic and diastolic blood pressures, GCS, intubation status, pulse rates, and respiratory rates. ISS, RTS, TRISS, and HLOS (hospital length of stay [LOS]) were calculated by the authors.

Patients were subsequently classified as either dead on arrival (DOA) or non‐DOA. DOA patients had either deceased on scene, during transportation, or immediately after arrival in the resuscitation room. Therefore, no further clinical investigation like imaging was done for these patients and their prognostic injury scores were not recorded. DOA patients were excluded from the analysis of clinical data and outcome prognostication. In 47 instances of non‐DOA patients, vital signs, GCS, and intubation status were not retrievable from the hospital database (26 in the prepandemic group and 21 in the pandemic group).

### 2.5 Statistical analysis

Continuous variables were compared by Student's *t*‐test (≥30 data points in each studied group rendered the use of nonparametric tests of hypothesis for continuous variables with non‐Gaussian distribution unnecessary). Categorical variables were analyzed by Pearson's *χ* ^2^ test. Data are reported as numbers, means and standard deviations (SD), medians and interquartile ranges (IQR), adjusted odds ratios (aOR), 95% confidence intervals (CI), and percentages as appropriate. A *p* Value of ≤0.05 is considered significant in the present study. Statistical analysis of data and generation of figures were done using Stata Statistical Software: Release 17: StataCorp LLC.

## 3 RESULTS

**Figure 1:**
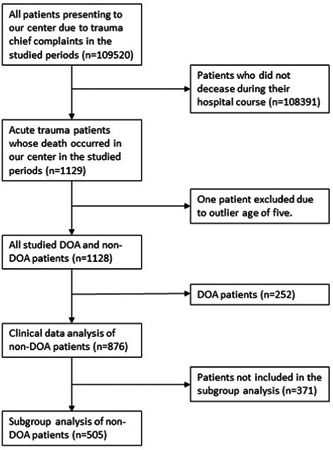
Patient flowchart detailing the inclusion and exclusion of patients at various stages of the analysis.

**Figure 2:**
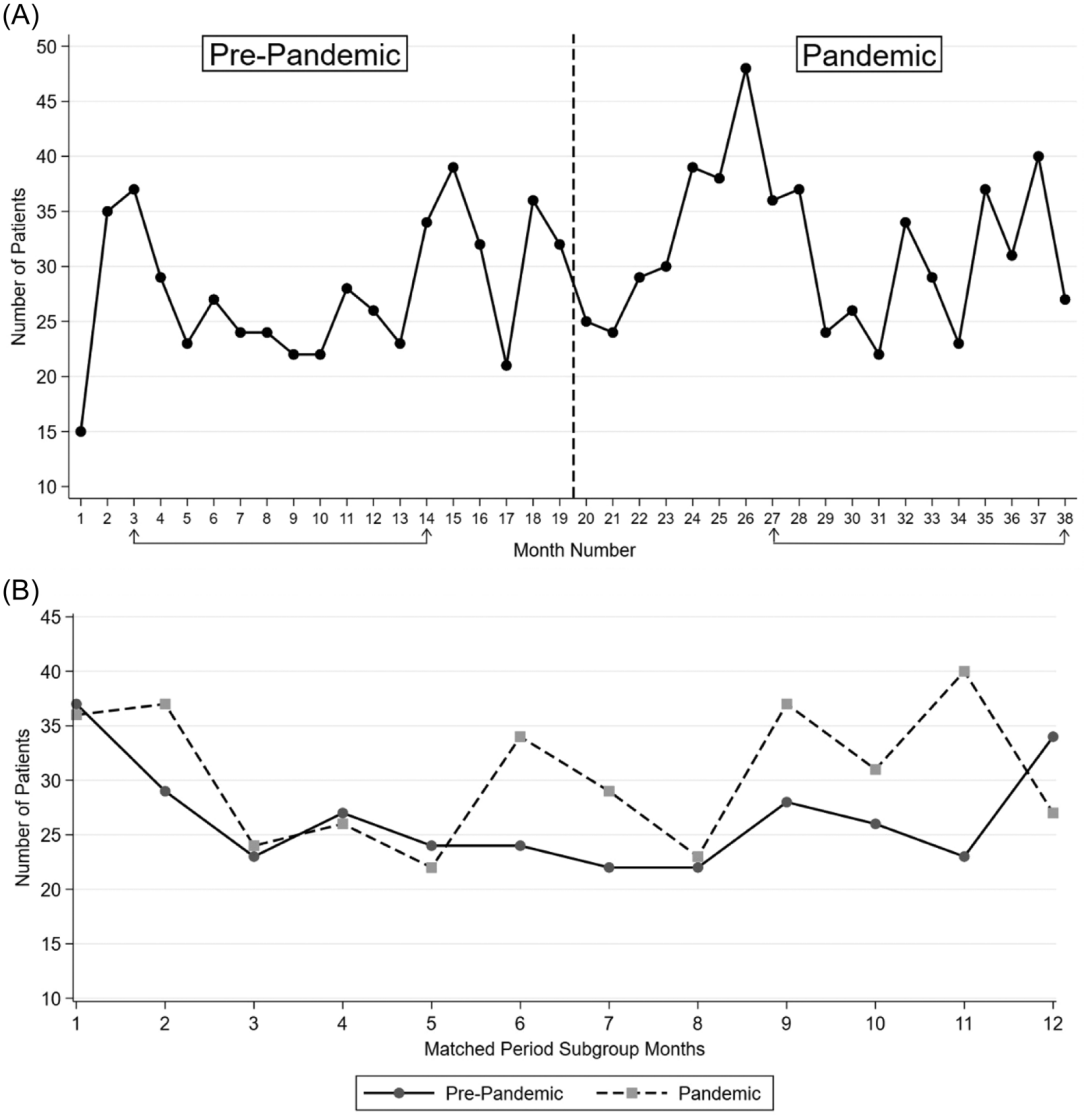
Number of trauma mortality patients. (A) in the two studied periods. (B) in the matched period subgroup months. The months indicated by lines under the x‐axis show the matched period subgroups.

In the stated study periods, a total of 118,263 patients presented to our referral trauma center regardless of the underlying causes and mechanism. According to our database, 8505 patients were admitted due to nontrauma‐related causes, and the admission indications of 288 patients were not clearly stated; therefore, these patients were excluded from the study. Figure 1 shows a flowchart detailing the inclusion and exclusion of patients at various stages in the analysis. The number of unique trauma‐related patients admitted to our center was 56,526 in the pre‐pandemic period and 52,944 in the pandemic period, exhibiting a decrease of 6.34%. The number of patients who passed away as a result of acute trauma and were included in our study was 1128. Out of these patients, 529 (46.81%) belonged to the prepandemic group and 599 (53.01%) to the pandemic group, showing a 13.23% upsurge. The crude mortality rates were 934 and 1129 per 100,000 trauma patients in prepandemic and pandemic periods respectively, showing a considerable discrepancy between the two periods (*p* Value calculated by Pearson's *χ* ^2^ test: 0.002). Figure 2 shows the number of trauma mortality patients in the two studied periods by month, and compares the number of patients in the matched period subgroup months. A detailed explanation of data in the matched period subgroup can be found in the subsequent subsection.

The total number of studied patients classified as DOA was 252, with 119 being in the pre‐pandemic group and the remaining 133 in the pandemic group, showing an 11.76% increase. A total of 106 DOA patients were in the matched period subgroup, with 47 belonging to the prepandemic and 59 to the pandemic groups. No statistically significant difference was detected between the two DOA patient groups regarding demographic features and mechanism of injury. Only the transportation type of these patients experienced a statistically significant change, with a considerable drop in the number of personal transportation and an increase in patients transferred from other facilities in the pandemic group. The analysis results of DOA patients are shown in Supporting Information: Table S1.

There were a total of 876 patients who died during the hospital course and were classified as non‐DOA, out of which 410 belonged to the prepandemic group and 466 to the pandemic group, demonstrating a 13.66% rise in numbers. In mechanisms of injury, no overall statistically significant difference can be shown, although, in the MVA subcategories, a statistically significant difference is noted, with an increase in motorcycle MVAs and a decrease in pedestrian MVAs. Similar to the pre‐pandemic period, in the pandemic period the largest percentage of mechanisms of injury consists of MVA (57.32% and 57.73%). The arrival mode of patients experienced a decrease in the number of personal transportation and a spike in the number of patients transferred from other facilities. Table 1 shows the demographic and epidemiological characteristics of non‐DOA patients.

**Table 1:** Demographic and epidemiological characteristics of patients classified as non‐DOA (*n* = 876).

Variable name	Overall (*n* = 876)	Prepandemic (*n* = 410)	Pandemic (*n* = 466)	*p* Value
Age, median (IQR)	58 (35−75)	59.5 (36−76)	56 (33−73)	0.073^a^
Age group, *n* (%)				0.597^b^
<18	28 (3.20%)	11 (2.68%)	17 (3.65%)	0.812^a^
18−65	496 (56.62%)	229 (55.85%)	267 (57.30%)	0.155^a^
>65	352 (40.18%)	170 (41.46%)	182 (39.06%)	0.107^a^
Gender				0.847^b^
Male, *n* (%)	664 (75.80%)	312 (76.10%)	312 (76.10%)	352 (75.54%)
Female, *n* (%)	212 (24.20%)	98 (23.90%)	114 (24.46%)	
Injury type				0.746^b^
Blunt, *n* (%)	853 (97.37%)	400 (97.56%)	453 (97.21%)	
Penetrating, *n* (%)	23 (2.63%)	10 (2.44%)	13 (2.79%)	
Mechanism of injury				0.197^b^
MVA, *n* (%)	504 (57.53%)	235 (57.32%)	269 (57.73%)	**0.016** b
Car, *n* (% of MVA)	170 (33.73%)	78 (33.19%)	92 (34.20%)	
Motorcycle, *n* (% of MVA)	125 (24.80%)	47 (20.00%)	78 (29.00%)	
Pedestrian, *n* (% of MVA)	202 (40.08%)	104 (44.26%)	98 (36.43%)	
Unspecified and Other, n (% of MVA)	7 (1.39%)	6 (2.55%)	1 (0.37%)	
Fall, n (%)	249 (28.42%)	108 (26.34%)	141 (30.26%)	0.098^b^
High, *n* (% of Falls)	76 (30.52%)	27 (25.00%)	49 (34.75%)	
Same level, *n* (% of Falls)	173 (69.48%)	81 (75.00%)	92 (65.25%)	
Firearms, *n* (%)	13 (1.48%)	7 (1.71%)	6 (1.29%)	
Stabbing, *n* (%)	10 (1.14%)	3 (0.73%)	7 (1.50%)	
Assault, *n* (%)	9 (1.03%)	7 (1.71%)	7 (0.43%)	
Environmental and other accidents, *n* (%)	12 (1.37%)	6 (1.46%)	6 (1.29%)	
Unspecified and unknown mechanism, *n* (%)	79 (9.02%)	44 (10.73%)	35 (7.51%)	
Arrival mode				**0.010** b
Primary EMS (Ambulance/HEMS), *n* (%)	589 (67.24%)	277 (67.56%)	312 (66.95%)	
Primary personal, *n* (%)	132 (15.07%)	74 (18.05%)	58 (12.45%)	
Transferred from other facilities, *n* (%)	155 (17.69%)	59 (14.39%)	96 (20.60%)	

*Note*: Percentages were calculated across the available data for each variable and column. Bold values are statistically significant at *p* < 0.05.

Abbreviations: EMS, emergency medical services; HEMS, helicopter emergency medical services; IQR, interquartile range; MVA, motor vehicle accident.

^a^ Student's t‐test.

^b^ Pearson's *χ* ^2^ test.

**Figure 3:**
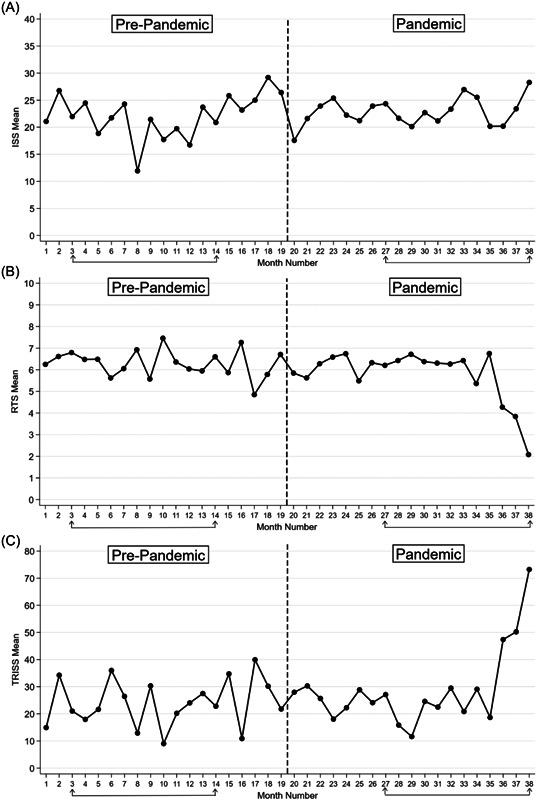
Mean trauma scores in each month of the studied period. (A) ISS. (B) RTS. (C) TRISS. The months indicated by lines under the x‐axis show the matched period subgroups.

In clinical features and prognosis, no significant variance is noted in the percentage of patients presenting with initial SBP less than 90 mmHg and prehospital intubation status. Regarding initial GCS, in both periods of study, the majority of patients were placed in the two groups of 3−5 (33.33% vs. 39.28%) and 13−15 (38.46% vs. 37.11%), although no statistically significant difference can be found. Patients in the pandemic period experienced a drop in severe thoracic injuries, with 48.05% of prepandemic patients scoring 3 or more in the related AIS compared to 40.34% of pandemic patients. The mean ± SD ISS in pre‐pandemic patients was 22.80 ± 13.57 (95% CI: 21.48−24.12), which compared to the mean ± SD of 22.91 ± 13.05 (95% CI: 21.73−24.10) in pandemic patients does not show a difference. The same applies to the means in different ISS range groups. The RTS mean ± SD in prepandemic and pandemic patients was respectively 6.32 ± 2.04 (95% CI: 6.074−6.58) and 5.82 ± 2.54 (95% CI: 5.55−6.10). Furthermore, the percentage of patients with an RTS of <4 surged in the pandemic period (10.04% vs. 16.22%). Mean TRISS was 23.97 (95% CI: 20.69−27.26) in the pre‐pandemic group and 28.93 (95% CI: 25.45−32.41) in the pandemic group. Additionally, a significantly higher percentage of patients in the pandemic group had a TRISS of ≥50 (16.60% vs. 25.23%). Figure 3 shows the mean ISS, RTS, and TRISS in each month of the studied period.

The mean hospital LOS of pandemic patients was meaningfully less than pre‐pandemic patients (15.57 vs. 12.54). A comparison of the number of patients passing away in ED, operating room (OR), and ICU in the two periods showed no significant change. These results are presented in Table 2.

**Table 2:** Initial clinical features, injury scores, and hospital course of patients classified as non‐DOA (*n* = 876).

Variable name	Overall (*n* = 876)	Prepandemic (*n* = 410)	Pandemic (*n* = 466)	*p* Value
Initial Systolic blood pressure < 90 (mmHg), *n* (%)*	179 (21.59%)	86 (22.40%)	93 (20.90%)	0.601^a^
Initial Pulse rate ≥ 100 (bpm), *n* (%)*	367 (44.27%)	166 (43.23%)	201 (45.17%)	0.575^a^
Prehospital intubation, *n* (%)*	228 (26.06%)	117 (28.54%)	111 (23.87%)	0.117^a^
GCS < 9, *n* (%)**	385 (50.26%)	170 (48.43%)	215 (51.81%)	0.352^a^
GCS group				0.322^a^
3−5, *n* (%)	280 (36.55%)	117 (33.33%)	163 (39.28%)	
6−8, *n* (%)	105 (13.71%)	53 (15.10%)	52 (12.53%)	
9−12, *n* (%)	92 (12.01%)	46 (13.11%)	46 (11.08%)	
13−15, *n* (%)	289 (37.73%)	135 (38.46%)	154 (37.11%)	
AIS category				
Head/neck ≥ 3, *n* (%)	459 (52.40%)	202 (49.27%)	257 (55.15%)	0.082^a^
Face ≥ 3, *n* (%)	23 (2.63%)	14 (3.41%)	9 (1.93%)	0.171^a^
Thorax ≥ 3, *n* (%)	385 (43.95%)	197 (48.05%)	188 (40.34%)	**0.022** a
Abdomen ≥ 3, *n* (%)	106 (12.10%)	43 (10.49%)	63 (13.52%)	0.170^a^
Extremity/pelvis ≥ 3, *n* (%)	239 (27.28%)	120 (29.27%)	119 (25.54%)	0.216^a^
External ≥ 3, *n* (%)	3 (0.34%)	3 (0.73%)	0 (0.00%)	0.064^a^
	22.86 ± 13.29 (876)	22.80 ± 13.57 (410)	22.91 ± 13.05 (466)	0.902^b^
ISS, mean ± SD (number)	22.86 ± 13.29 (876)	22.80 ± 13.57 (410)	22.91 ± 13.05 (466)	0.902^b^
ISS group				**0.022** a
<16, *n* (%)	262 (29.91%)	119 (29.02%)	143 (30.69%)	0.914^b^
16‐24, *n* (%)	197 (22.49%)	109 (26.59%)	88 (18.88%)	0.519^b^
>24, *n* (%)	471 (47.60%)	182 (44.39%)	235 (50.43%)	0.168^b^
RTS, mean ± SD (number)	6.04 ± 2.35 (592)	6.32 ± 2.04 (259)	5.82 ± 2.54 (333)	**0.008** b
RTS < 4, *n* (%)	80 (13.51%)	26 (10.04%)	54 (16.22%)	**0.029** a
TRISS, mean ± SD (number)	26.76 ± 29.19 (592)	23.97 ± 26.83 (259)	28.93 ± 32.26 (333)	**0.046** b
TRISS ≥ 50%, *n* (%)	127 (21.45%)	43 (16.60%)	84 (25.23%)	**0.011** a
Hospital LOS (days), median (IQR)	13.96 (1−20)	15.57 (1−21)	12.54 (1−17)	**0.034** b
Death in ED, *n* (%)	37 (4.22%)	22 (5.37%)	15 (3.22%)	0.115^a^
Death in OR, *n* (%)	60 (6.85%)	22 (5.37%)	38 (8.15%)	0.103^a^
Death in ICU, *n* (%)	748 (85.39%)	348 (84.88%)	400 (85.84%)	0.688^a^

*Note*: Percentages were calculated across the available data for each variable and column; *Number of available data instances: 829; **Number of available data instances: 820. Bold values are statistically significant at *p* < 0.05.

Abbreviations: AIS, abbreviated injury score; CI, confidence interval; DOA, dead on arrival; ED, emergency department; GCS, Glasgow coma scale; ICU, intensive care unit; IQR, interquartile range; ISS, injury severity score; LOS, length of stay; OR, operating room; PR, pulse rate; RTS, revised trauma score; SBP, systolic blood pressure; SD, standard deviation; TRISS, trauma and injury severity scoring.

^a^ Pearson's *χ* ^2^ test.

^b^ Student's *t*‐test;

### 3.1 Matched period subgroup analysis

The number of mortality patients in the matched period subgroup was 285 in the prepandemic group and 366 in the pandemic group, making a total of 651. Figure 2 shows the number of trauma mortality patients in the matched period subgroup months. In the matched period subgroup, there were 213 non‐DOA patients in the pre‐pandemic group and 292 in the pandemic group, which shows a 37.10% increase. Analysis of the matched period subgroup was done and yielded increases in mean ISS and TRISS, a decrease in mean RTS, and an increase in the percentage of patients with TRISS ≥ 50% (*p* ≤ 0.05). No change was noted in patient age in the matched period subgroup. The subgroup analysis is shown in Table 3.

**Table 3:** Matched period subgroup analysis of patients classified as non‐DOA (*n* = 505).

	Overall (*n* = 505)	Prepandemic (*n* = 213)	Pandemic (*n* = 292)	*p* Value
Age, mean ± SD	56.17 ± 23.40	58.26 ± 23.15	54.64 ± 23.50	0.085^a^
ISS, mean ± SD (number)	22.09 ± 13.31 (505)	20.60 ± 13.17 (213)	23.18 ± 13.33 (292)	**0.031** a
ISS ≥ 16, *n* (%)	344 (68.12%)	141 (66.20%)	203 (69.52%)	0.429^b^
RTS, mean ± SD (number)	5.90 ± 2.43 (339)	6.35 ± 1.88 (134)	5.61 ± 2.70 (205)	**0.003** a
RTS < 4, *n* (%)	53 (15.63%)	15 (11.19%)	38 (18.54%)	0.069^b^
TRISS, mean ± SD (number)	28.02 ± 31.71 (339)	22.65 ± 26.39 (134)	31.53 ± 34.36 (205)	**0.007** a
TRISS, median (IQR)	12.18 (3.76‐41.22)	10.60 (3.83‐31.11)	12.35 (3.76‐57.36)	
TRISS ≥ 50%, *n* (%)	78 (23.01%)	20 (14.93%)	58 (28.29%)	**0.004** b

*Note*: Bold values are statistically significant at *p* < 0.05.

Abbreviations: DOA, dead on arrival; IQR, interquartile range; ISS, injury severity score; RTS, revised trauma score; SD, standard deviation; TRISS, trauma and injury severity scoring.

^a^ Student's t‐test.

^b^ Pearson's *χ* ^2^ test.

## 4 DISCUSSION

COVID‐19 was formally recognized in Iran in February 2020 and as of the time of writing this article (May 2023), has claimed the lives of 146,295 persons and gone through more than six disease peaks according to the latest official statistics declared by the Islamic Republic of Iran Ministry of Health and Medical Education.^34^ Multiple stay‐at‐home orders and public disease mitigation policies were enacted that have been shown to change the epidemiology and mechanisms of injuries.^35^ The Healthcare system in Iran experienced increasing workload and decreasing revenue,^36^, ^37^ which are factors that can potentially decrease the quality of care provided.

Our results have shown that despite restrictions (ranging from light to severe) on community mobility, the demographic features of the trauma mortality patient population did not undergo any transformations during the COVID‐19 pandemic. The age and gender distribution of patients remained unchanged. Major traumatic injury mechanisms are similar in the distribution in the two studied periods, with MVAs being the lead culprit mechanism. An increase in the number of motorcycle accidents was noted. The official MVA statistics provided by the Iranian Legal Medicine Organization reported national decreases of 9.1% in deaths and 20.3% in nonfatal injuries resulting from MVAs in the first year after the pandemic, with respective 9.0% and 14.6% increases in the next year. These fluctuations are rather different in the region in which our referral trauma center is located (Fars province), with decreases of 2.2% in deaths and 20.4% in nonfatal injuries in the first year after the pandemic, and respective 11.9% and 14.2% increases in the next year,^38^ which puts the regional trend in MVA deaths in the upward direction. The same initial decrease and subsequent bounce‐back pattern can be seen in the local new year holidays (around 15 March to 5 April, which is typically a surge period for MVAs), with a national decrease of 41.6% in MVA deaths during the first holiday period after the start COVID‐19 (regional: 15.4% decrease) and a national increase 92.6% in the next (regional: 114.3%).^39^

Multiple studies examining trauma during COVID‐19 have reported a drop in the number of minor injuries and a reduction in MVAs.^40^, ^41^, ^42^, ^43^, ^44^, ^45^ Despite this, studies focusing on patients with more severe injuries have reported that severe injury mechanisms in the COVID‐19 era remain similar to pre‐COVID‐19 era; our study includes only mortality patients, a population who naturally had sustained more severe injuries and its findings are consistent with previously published studies focusing on severe trauma.^15^, ^46^, ^47^

The arrival mode of patients to our center showed significant changes in the pandemic period, with a higher number being transported by EMS/HEMS and transferred from other facilities, and a lower number by personal vehicles and means. Several lower‐level trauma centers were fully or partially converted to COVID‐19 centers at different points during the pandemic,^48^ but the level 1 trauma center where this study was conducted did not receive any primarily COVID‐19 patients. The changes in arrival mode reflect both the poorer condition of patients in the pandemic group and a spillover effect from other centers, which potentially delays life‐saving treatment.

Considering the retrospective clinical picture of admitted mortality non‐DOA patients, an overall poorer prognosis was established and demonstrated by the decreased RTS mean, a larger number of patients with RTS < 4, increased TRISS mean, and an increased number of patients with TRISS ≥ 50%. The worse clinical prognosis and condition of patients during the height of the pandemic is further grounded in the matched period subgroup analysis results, which showed poorer prognosis in the pandemic group across ISS, RTS, and TRISS, plus an increased percentage of patients with TRISS ≥ 50%. In AIS ≥ 3 categories, only thoracic injuries demonstrated a statistically significant decrease in the pandemic period. These findings imply that although the age of patients, major trauma mechanisms (blunt vs. penetrating), and anatomic patterns of injuries were largely unaltered in the pandemic period, patients had a generally poorer initial physiologic status (instituted by our findings regarding RTS) which contributed to their higher mean TRISS and the overall higher predicted mortality probability and recorded mortality numbers. This realization warrants further investigations into our trauma system to determine the potential causes of these outcomes. Stronger results of the subgroup analysis in favor of poorer patient conditions suggest that in the initial months of the pandemic, the epidemiology and severity of injuries were pointedly different from the months of the pandemic that followed. This difference can be explained by the aforementioned initial decrease in fatal MVAs and the ensuing bounce‐back and also its upward trend, considering MVAs comprise the majority of trauma mortality in our study.

Poorer physiologic condition and lower RTS can potentially be due to reduced quality of care in pre‐hospital settings, increased transport time due to increased EMS workload,^49^, ^50^ and increased number of patient transfers and referrals, although this subject remains understudied and reports of no significant pre‐hospital transport time have been made in other countries.^51^ Pandemic‐related strain on healthcare resources may have led to delays and unavailability of trauma care, impacting patients' physiologic conditions. Supporting this, we observed an increase in transfers from other centers during the pandemic, potentially due to reallocation or care capacity being undercut, which subsequently resulted in worsened patient conditions when arrived at our center.

A similar number of patients passed away in the ED, OR, and ICU in the two periods, which reflects the hospital disposition workflow for severely injured patients being unaffected by pandemic conditions. Occult and confirmed concurrent COVID‐19 in some patients can be a potential cause of poorer physiologic conditions in the pandemic group, but no data regarding the COVID‐19 status of patients was available to the authors.

Several limitations to our study can be pointed out. We only included trauma mortality patients, therefore excluding a large number of minor nonfatal traumas and this greatly affects our injury mechanisms results. Our center primarily receives adult patients and as a result, pediatric trauma patients are mostly excluded (although a small number of pediatric patients all older than 10 were admitted to our center and included in our study). In several instances, missing data did not allow for the calculation of RTS and TRISS. RTS and TRISS exclude patients who arrive at the ED intubated, and considering intubated patients are generally of poorer overall condition and have experienced more severe trauma, the aggregate RTS and TRISS can underestimate trauma severity in the group by excluding intubated patients. Another probable bias and imprecision can be using the regression coefficients from MTOS 1995^33^ that were developed in a different sociocultural environment and may not be completely compatible with our trauma epidemiology and patient population. Other inherent limitations of these trauma scores also apply to our study. No data regarding pre‐hospital transport times were available which is an important factor influencing the clinical status of our patients. Finally as mentioned before, COVID‐19 in some patients can be a potential cause of poorer physiologic conditions in the pandemic group, the data on which were not available to us.

### 4.1 Limitations

The COVID‐19 pandemic hit Iran especially brutally, with a high number of cases and mortality per capita, delay in mass vaccination, a high number of COVID‐19‐related deaths among physicians, and economic hardships.^52^ Our findings can be interpreted that despite Shahid Rajaei (Emtiaz) Hospital and Fars province healthcare system faced difficulties on multiple fronts, the increased mortality was the result of the poorer clinical status of these patients during the pandemic period and general fatal MVA trends. These findings are mostly attributable to level 1 trauma centers that do not receive non‐trauma COVID‐19 patients. However, lower‐level trauma centers also seem relevant to understanding the impact of the pandemic on trauma care. These hospitals were the ones that were mainly repurposed into COVID‐19 care, hence they may have experienced decreased quality of care or increased referrals. Mortality in trauma cases is influenced by numerous confounding factors, and the unique characteristics of the Iranian healthcare system should also be considered. Hence, the authors very cautiously suggest generalizability to level 1 trauma centers in low‐resource settings, but applicability must be evaluated within each specific context. The authors would like to highlight that further multicenter research (including lower‐level centers) and synthesized evidence is required to better understand the impact of the COVID‐19 pandemic on trauma care and to pinpoint the causes of poorer clinical prognosis in patients admitted during the pandemic period.

### 4.2 Optimizing trauma care during pandemics in middle and low‐income settings

In light of the challenges posed by major pandemics, particularly in low and middle‐income countries with limited healthcare resources, it is imperative to consider potential solutions and recommendations to optimize trauma care. The fragility of health systems in these settings makes them more susceptible to collapse during crises, such as the COVID‐19 pandemic, and raises concerns about their adaptability to future pandemics.

Strengthening pre‐hospital and emergency medical services can significantly improve the timely and effective delivery of trauma care. Investing in training, infrastructure, and communication systems for first responders is essential.

Proactive preparation, including the development of comprehensive pandemic response plans, can ensure that trauma care remains a prioritized and well‐supported component of healthcare delivery.

Having a dedicated trauma center with a strategic allocation of trauma beds, separate from pandemic patient care, is crucial. This approach ensures that trauma patients receive specialized attention without compromising their care during pandemic surges.

Developing flexible triage systems that can be rapidly adjusted based on the evolving needs of a pandemic is essential. These systems should consider the dynamic nature of patient loads and allocate resources accordingly. Promoting data sharing among healthcare institutions can enhance collective knowledge and help attain dynamic resource allocation.

## 5 CONCLUSIONS

We studied trauma patients who experienced in‐hospital mortality in two time periods of equal length before and after the start of the COVID‐19 pandemic. The mortality of patients admitted to our center increased during the pandemic period. No changes in demographics and injury mechanisms of these patients were found. In patients included in our study, the overall clinical prognosis estimated by trauma scores was significantly worse during the pandemic period. The poorer prognosis was likely primarily associated with poorer physiological conditions, as the scores that incorporated physiological parameters displayed more pronounced deterioration during the pandemic period. Further research is required to accurately delineate the causes of increased mortality during the pandemic period.

## AUTHOR CONTRIBUTIONS

**Seyyed HamidReza Ayatizadeh**: Data curation; formal analysis; writing—original draft. **Roham Borazjani**: Conceptualization; data curation; supervision; validation. **Reza Fereidooni**: Validation; writing—review and editing. **Kazem Jamali**: Conceptualization; validation; visualization. **Hossein Abdolrahimzadeh Fard**: Conceptualization; methodology; validation. **Reza Homaeifar**: Data curation; methodology; validation. **Leila Shayan**: Data curation. **Zohreh Saadatjoo**: Data curation. **Shahram Paydar**: Conceptualization; funding acquisition; project administration; resources; supervision.

## CONFLICT OF INTEREST STATEMENT

The authors declare no conflict of interest.

## ETHICS STATEMENT

All methods were performed in accordance with the ethical standards as laid down in the Declaration of Helsinki and its later amendments or comparable ethical standards. Approval was granted by Shiraz University of Medical Sciences ethics committee (code: IR.SUMS.MED.REC.1401.305). The ethical committee (IRB) of Shiraz University of Medical Sciences waived the need to obtain participants' or their authorized representative's informed consent.

## TRANSPARENCY STATEMENT

The lead author Reza Fereidooni affirms that this manuscript is an honest, accurate, and transparent account of the study being reported; that no important aspects of the study have been omitted; and that any discrepancies from the study as planned (and, if relevant, registered) have been explained.

## Supporting information

Supporting information.

## Data Availability

The data set supporting the conclusions of this article is available for academic researchers via the research deputy of Shiraz Medical School (med_thesis@sums.ac.ir) upon reasonable request.

## References

[1] Cucinotta D , Vanelli M . WHO declares COVID‐19 a pandemic. Acta bio‐medica. 2020;91(1):157‐160. 32191675 10.23750/abm.v91i1.9397 PMC7569573

[2] Sabetkish N , Rahmani A . The overall impact of COVID ‐19 on healthcare during the pandemic: a multidisciplinary point of view. Health Sci Rep. 2021;4(4):e386. 34622020 10.1002/hsr2.386 PMC8485600

[3] McGuinness MJ , Hsee L . Impact of the COVID‐19 national lockdown on emergency general surgery: Auckland City Hospital's experience. ANZ J Surg. 2020;90(11):2254‐2258. 32940409 10.1111/ans.16336

[4] Balogh ZJ , Way TL , Hoswell RL . The epidemiology of trauma during a pandemic. Injury. 2020;51(6):1243‐1244. 32540093 10.1016/j.injury.2020.05.039 PMC7292592

[5] Haut ER , Leeds IL , Livingston DH . The effect on trauma care secondary to the COVID‐19 pandemic: collateral damage from diversion of resources. Ann Surg. 2020;272(3):e204‐7. 32452950 10.1097/SLA.0000000000004105 PMC7467027

[6] Bram JT , Johnson MA , Magee LC , et al. Where have all the fractures gone? the epidemiology of pediatric fractures during the COVID‐19 pandemic. J Ped Orthop. 2020;40(8):373‐379. 10.1097/BPO.0000000000001600 32433260

[7] Maryada VR , Mulpur P , Guravareddy AV , Pedamallu SK , Vijay Bhasker B . Impact of COVID‐19 pandemic on orthopaedic trauma volumes: a multi‐centre perspective from the state of telangana. Indian J Orthop. 2020;54(2):368‐373. 32836367 10.1007/s43465-020-00226-z PMC7423500

[8] Vos T , Lim, SS , Abbafati C , et al. Global burden of 369 diseases and injuries in 204 countries and territories, 1990–2019: a systematic analysis for the global burden of disease study 2019. Lancet. 2020;396(10258):1204‐1222. 33069326 10.1016/S0140-6736(20)30925-9 PMC7567026

[9] Reynolds TA , Stewart B , Drewett I , et al. The impact of trauma care systems in low‐ and middle‐income countries. Annu Rev Public Health. 2017;38:507‐532. 28125389 10.1146/annurev-publhealth-032315-021412

[10] Waseem S , Nayar SK , Hull P , et al. The global burden of trauma during the COVID‐19 pandemic: a scoping review. J Clin Orthop Trauma. 2021;12(1):200‐207. 33223749 10.1016/j.jcot.2020.11.005 PMC7666557

[11] Yasin YJ , Grivna M , Abu‐Zidan FM . Global impact of COVID‐19 pandemic on road traffic collisions. World J Emerg Surg. 2021;16(1):51. 34583713 10.1186/s13017-021-00395-8 PMC8478263

[12] Giudici R , Lancioni A , Gay H , et al. Impact of the COVID‐19 outbreak on severe trauma trends and healthcare system reassessment in Lombardia, Italy: an analysis from the regional trauma registry. World J Emerg Surg. 2021;16(1):39. 34281575 10.1186/s13017-021-00383-y PMC8287111

[13] Devarakonda AK , Wehrle CJ , Chibane FL , Drevets PD , Fox ED , Lawson AG . The effects of the COVID‐19 pandemic on trauma presentations in a level one trauma center. Am Surg. 2021;87(5):686‐689. 33231483 10.1177/0003134820973715 PMC7688434

[14] Van Aert GJJ , Van Der Laan L , Boonman‐De Winter LJM , et al. Effect of the COVID‐19 pandemic during the first lockdown in the Netherlands on the number of trauma‐related admissions, trauma severity and treatment: the results of a retrospective cohort study in a level 2 trauma centre. BMJ Open. 2021;11(2):e045015. 10.1136/bmjopen-2020-045015 PMC7898225 33608406

[15] Riuttanen A , Ponkilainen V , Kuitunen I , Reito A , Sirola J , Mattila VM . Severely injured patients do not disappear in a pandemic: incidence and characteristics of severe injuries during COVID‐19 lockdown in Finland. Acta Orthop. 2021;92(3):249‐253. 33538233 10.1080/17453674.2021.1881241 PMC8231355

[16] Crenn V , El Kinani M , Pietu G , et al. Impact of the COVID‐19 lockdown period on adult musculoskeletal injuries and surgical management: a retrospective monocentric study. Sci Rep. 2020;10(1):22442. 33384443 10.1038/s41598-020-80309-x PMC7775434

[17] Adiamah A , Thompson A , Lewis‐Lloyd C , et al. The ICON trauma study: the impact of the COVID‐19 lockdown on major trauma workload in the UK. Euro J Trauma Emerg Surg. 2021;47(3):637‐645. 10.1007/s00068-020-01593-w PMC7871318 33559697

[18] Driessen MLS , Sturms LM , Bloemers FW , et al. The detrimental impact of the COVID‐19 pandemic on major trauma outcomes in the Netherlands. Ann Surg. 2022;275(2):252‐258. 35007227 10.1097/SLA.0000000000005300 PMC8745885

[19] Gallaher JR , Yohann A , Kajombo C , Schneider A , Purcell L , Charles A . Reallocation of hospital resources during COVID‐19 pandemic and effect on trauma outcomes in a resource‐limited setting. World J Surg. 2022;46:2036‐2044. 35754058 10.1007/s00268-022-06636-4 PMC9244557

[20] Hornor MA , Hoeft C , Nathens AB . Quality benchmarking in trauma: from the NTDB to TQIP. Current Trauma Reports. 2018;4(2):160‐169.

[21] Domingues, CdA , Coimbra, R , Poggetti RS , et al. New trauma and injury severity score (TRISS) adjustments for survival prediction. World J Emerg Surg. 2018;13(1):1‐6. 29541155 10.1186/s13017-018-0171-8 PMC5840784

[22] Halvachizadeh S , Baradaran L , Cinelli P , Pfeifer R , Sprengel K , Pape HC . How to detect a polytrauma patient at risk of complications: a validation and database analysis of four published scales. PLoS One. 2020;15(1):e0228082. 31978109 10.1371/journal.pone.0228082 PMC6980592

[23] Eichelberger MR , Bowman LM , Sacco WJ , Mangubat EA , Lowenstein AD , Gotschall CS . Trauma score versus revised trauma score in TRISS to predict outcome in children with blunt trauma. Ann Emerg Med. 1989;18(9):939‐942. 2764326 10.1016/s0196-0644(89)80457-3

[24] Von Elm E , Altman DG , Egger M , Pocock SJ , Gøtzsche PC , Vandenbroucke JP . The strengthening the reporting of observational studies in epidemiology (STROBE) statement: guidelines for reporting observational studies. PLoS Med. 2007;4(10):e296. 17941714 10.1371/journal.pmed.0040296 PMC2020495

[25] Gabbe BJ , Cameron PA , Wolfe R . TRISS: does it get better than this? Acad Emerg Med. 2004;11(2):181‐186. 14759963

[26] Greenspan L , McLellan BA , Greig H . Abbreviated injury scale and injury severity score: a scoring chart. J Trauma. 1985;25(1):60‐64. 3965737 10.1097/00005373-198501000-00010

[27] Civil ID , Schwab CW . The abbreviated injury scale, 1985 revision: a condensed chart for clinical use. J Trauma. 1988;28(1):87‐90. 3339667 10.1097/00005373-198801000-00012

[28] Baker SP , OʼNEILL B , Haddon W , Long WB . The injury severity score: a method for describing patients with multiple injuries and evaluating emergency care. J Trauma. 1974;14(3):187‐196. 4814394

[29] Beverland DE , Rutherford WH . An assessment of the validity of the injury severity score when applied to gunshot wounds. Injury. 1983;15(1):19‐22. 6885141 10.1016/0020-1383(83)90156-0

[30] Champion HR , Sacco WJ , Copes WS , GANN DS , GENNARELLI TA , FLANAGAN ME . A revision of the trauma score. J Trauma. 1989;29(5):623‐629. 2657085 10.1097/00005373-198905000-00017

[31] Boyd CR , Tolson MA , Copes WS . Evaluating trauma care: the TRISS method. J Trauma. 1987;27(4):370‐378. 3106646

[32] Hosseinpour R , Barghi A , Mehrabi S , Salaminia S , Tobeh P . Prognosis of the trauma patients according to the trauma and injury severity score (TRISS); A diagnostic accuracy study. Bull Emerg Trauma. 2020;8(3):148‐155. 32944574 10.30476/BEAT.2020.84613 PMC7468220

[33] Champion HR , Sacco WJ , Copes WS . Injury severity scoring again. J Trauma. 1995;38(1):94‐95. 7745669 10.1097/00005373-199501000-00024

[34] Goverment of the Islamic Republic of Iran, Ministry of Health and Medical Education. Health Ministry's Updates on COVID‐19. Accessed May 17, 2022. https://irangov.ir/detail/414837

[35] Rassouli M , Ashrafizadeh H , Shirinabadi Farahani A , Akbari ME . COVID‐19 management in Iran as one of the most affected countries in the world: advantages and weaknesses. Front Public Health. 2020;8:510. 33072688 10.3389/fpubh.2020.00510 PMC7533538

[36] Behzadifar M , Aalipour A , Kehsvari M , et al. The effect of COVID‐19 on public hospital revenues in Iran: an interrupted time‐series analysis. PLoS One. 2022;17(3):e0266343. 35358279 10.1371/journal.pone.0266343 PMC8970352

[37] Shoja E , Aghamohammadi V , Bazyar H , et al. Covid‐19 effects on the workload of Iranian healthcare workers. BMC Public Health. 2020;20(1):1636. 33138798 10.1186/s12889-020-09743-w PMC7605333

[38] Organization ILM . MVA statistics. Accessed April 3, 2022. https://www.lmo.ir/web_directory/53999-%D8%AA%D8%B5%D8%A7%D8%AF%D9%81%D8%A7%D8%AA.html

[39] Organization ILM . Nowruz MVA deaths. Accessed April 3, 2022. https://www.lmo.ir/web_directory/53998-%D8%AA%D8%B5%D8%A7%D8%AF%D9%81%D8%A7%D8%AA-%D9%86%D9%88%D8%B1%D9%88%D8%B2%DB%8C.html

[40] Williams CH , Scott EM , Dorfman JD , Simon BJ . Traumatic injury under COVID‐19 stay‐at‐home advisory: experience of a new england trauma center. J Surg Res. 2022;269:165‐170. 34563843 10.1016/j.jss.2021.08.005 PMC8352668

[41] Venter A , Lewis CM , Saffy P , Chadinha LP . Locked down: impact of COVID‐19 restrictions on trauma presentations to the emergency department. SAMJ. 2020;111(1):52‐56. 33404006 10.7196/SAMJ.2021.v111i1.15289

[42] Sherman WF , Khadra HS , Kale NN , Wu VJ , Gladden PB , Lee OC . How did the number and type of injuries in patients presenting to a regional level I trauma center change during the COVID‐19 pandemic with a stay‐at‐home order? Clin Orthop Rel Res. 2021;479(2):266‐275. 10.1097/CORR.0000000000001484 PMC7899709 32969846

[43] Biswas S , Rhodes H , Petersen K . 328: pandemic paradox: patterns of traumatic injuries during COVID‐19 despite the safe haven of home. Crit Care Med. 2021;49(1):152.

[44] Matthay ZA , Kornblith AE , Matthay EC , et al. The DISTANCE study: determining the impact of social distancing on trauma epidemiology during the COVID‐19 epidemic—an interrupted time‐series analysis. J Trauma Acute Care Surg. 2021;90(4):700‐707. 33252457 10.1097/TA.0000000000003044 PMC7979514

[45] Rhodes HX , Petersen K , Biswas S . Trauma trends during the initial peak of the COVID‐19 pandemic in the midst of lockdown: experiences from a rural trauma center. Cureus. 2020;12(8):e9811. 10.7759/cureus.9811 32953322 PMC7494409

[46] Fuchs KF , Eden L , Gilbert F , et al. Führt eine COVID‐19‐bedingte ausgangsbeschränkung zu einer reduktion schwer verletzter patienten an einem überregionalen traumazentrum? Unfallchirurg. 2021;124(5):352‐357. 33252703 10.1007/s00113-020-00924-1 PMC7702725

[47] Trier F , Fjølner J , Raaber N , Sørensen AH , Kirkegaard H . Effect of the COVID‐19 pandemic at a major Danish trauma center in 2020 compared with 2018–2019: a retrospective cohort study. Acta Anaesth Scand. 2022;66(2):265‐272. 34748218 10.1111/aas.13997 PMC8653017

[48] Jamaati H , Dastan F , Esmaeili Dolabi S , et al. COVID‐19 in Iran: a model for crisis management and current experience. Iranian J Pharmace Res. 2020;19(2):1‐8. 10.22037/ijpr.2020.113365.14255 PMC7667532 33224206

[49] Saberian P , Conovaloff J , Vahidi E , Hasani‐Sharamin P , Kolivand PH . How the COVID‐19 epidemic affected prehospital emergency medical services in Tehran, Iran. West J Emerg Med. 2020;21(6):110‐116. 10.5811/westjem.2020.8.48679 33052824 PMC7673868

[50] Mohammadi F , Tehranineshat B , Bijani M , Khaleghi AA . Management of COVID‐19‐related challenges faced by EMS personnel: a qualitative study. BMC Emerg Med. 2021;21(1):95. 34391404 10.1186/s12873-021-00489-1 PMC8363870

[51] Jarvis S , Salottolo K , Berg GM , et al. Examining emergency medical services' prehospital transport times for trauma patients during COVID‐19. Am J Emerg Med. 2021;44:33‐37. 33578329 10.1016/j.ajem.2021.01.091 PMC7857109

[52] Abdoli A . Iran, sanctions, and the COVID‐19 crisis. J Med Econ. 2020;23(12):1461‐1465. 33249954 10.1080/13696998.2020.1856855

