# Supplementary material for: Impact of COVID‐19 pandemic on trauma mortality patients: A retrospective observational study in an Iranian level 1 trauma center
Source: Health Sci Rep. 2024 Feb 13;7(2):e1883. doi: 10.1002/hsr2.1883 (PMC10864731; doi:10.1002/hsr2.1883)
Supplement: Supplementary file 1 — Supporting information. [file HSR2-7-e1883-s001.docx]

| Table S1. Demographic and epidemiological characteristics of patients classified as DOA (n=252). | | | | |
| --- | --- | --- | --- | --- |
| Variable Name | **Overall**  **(n=252)** | **Pre-Pandemic (n=119)** | **Pandemic (n=133)** | **P value** |
| Age, mean ±SD | 41.84 ±20.10 | 41.97 ±20.27 | 41.71 ±20.02 | 0.918 ^a^ |
| Age, median (IQR) | 38 (25-58.5) | 37 (25-58) | 38 (24-59) |  |
| Age, range | 11-94 | 15-94 | 11-94 |  |
| Age group, n (%) |  |  |  | 0.624 ^b^ |
| - <18 | 14 (5.56%) | 6 (5.04%) | 8 (6.02%) | 0.134 ^a^ |
| - 18-65 | 197 (78.17%) | 94 (78.99%) | 103 (77.44%) | 0.764 ^a^ |
| - >65 | 41 (16.27%) | 19 (15.97%) | 22 (16.54%) | 0.079 ^a^ |
| Gender, n (%) |  |  |  | 0.805 ^b^ |
| - Male | 207 (82.14%) | 97 (81.51%) | 110 (82.71%) |  |
| - Female | 45 (17.86%) | 22 (18.49%) | 23 (17.29%) |  |
| Injury type |  |  |  | 0.623 ^b^ |
| - Blunt, n (%) | 233 (92.46%) | 109 (91.60%) | 124 (93.23%) |  |
| - Penetrating, n (%) | 19 (7.54%) | 10 (8.40%) | 9 (6.77%) |  |
| Mechanism of injury |  |  |  | 0.305 ^b^ |
| MVA, n (%) | 180 (75.69%) | 76 (68.97%) | 104 (81.50%) | 0.628 ^b^ |
| - - Car, n (% of MVA) | 88 (48.89%) | 41 (53.95%) | 47 (45.19%) |  |
| - - Motorcycle, n (% of MVA) | 37 (20.56%) | 13 (17.11%) | 24 (23.08%) |  |
| - - Pedestrian, n (% of MVA) | 51 (28.33%) | 20 (26.32%) | 31 (29.81%) |  |
| - - Unspecified and Other, n (% of MVA) | 4 (2.22%) | 2 (2.63%) | 2 (1.92%) |  |
| - Fall, n (%) | 35 (8.83%) | 22 (11.98%) | 13 (6.11%) | 0.968 ^b^ |
| - - High, n (% of Falls) | 19 (54.29%) | 12 (54.55%) | 7 (53.85%) |  |
| - - Same Level, n (% of Falls) | 16 (45.71%) | 10 (45.45%) | 6 (46.15%) |  |
| - Firearms, n (%) | 9 (3.03%) | 5 (3.63%) | 4 (2.51%) |  |
| - Stabbing, n (%) | 10 (5.05%) | 5 (5.44%) | 5 (4.70%) |  |
| - Assault, n (%) | 3 (0.25%) | 2 (0.36%) | 1 (0.16) |  |
| - Environmental and Other Accidents, n (%) | 4 (0.67%) | 2 (0.73%) | 2 (0.63%) |  |
| - Unspecified and Unknown Mechanism, n (%) | 11 (6.48%) | 7 (8.89%) | 4 (4.39%) |  |
| Arrival mode |  |  |  | **0.005** ^b^ |
| - Primary EMS (Ambulance/HEMS), n (%) | 220 (87.30%) | 104 (87.39%) | 116 (87.22%) |  |
| - Primary Personal, n (%) | 18 (7.14%) | 13 (10.92%) | 5 (3.76%) |  |
| - Transferred From Other Facilities, n (%) | 14 (5.56%) | 2 (1.68%) | 12 (9.02%) |  |
| DOA dead on arrival; SD standard deviation; IQR interquartile range; MVA motor vehicle accident; EMS emergency medical services; HEMS helicopter emergency medical services; ^a^ Student’s t-test; ^b^ Pearson’s chi-squared test; Percentages were calculated across the available data for each variable and column. | | | | |
